# Unveiling the potential of HSPA4: a comprehensive pan-cancer analysis of HSPA4 in diagnosis, prognosis, and immunotherapy

**DOI:** 10.18632/aging.205496

**Published:** 2024-01-31

**Authors:** Junhao Yang, Xiaoxiao Wu, Jianhong You

**Affiliations:** 1 Department of Surgical Oncology and General Surgery, The First Hospital of China Medical University, Shenyang 110001, China; 2 Department of Rheumatology, Second Affiliated Hospital, College of Medicine, Zhejiang University, Hangzhou 310000, China; 3 Department of Ultrasound, Zhongshan Hospital of Xiamen University, School of Medicine, Xiamen University, Xiamen 361000, China

**Keywords:** HSPA4, pan-cancer, bioinformatics, immunotherapy, prognosis

## Abstract

With the global rise in cancer incidence and mortality rates, research on the topic has become increasingly urgent. Among the significant players in this field are heat shock proteins (HSPs), particularly HSPA4 from the HSP70 subfamily, which has recently garnered considerable interest for its role in cancer progression. However, despite numerous studies on HSPA4 in specific cancer types, a comprehensive analysis across all cancer types is lacking. This study employs various bioinformatics techniques to delve into the role of HSPA4 in pan-cancer. Our objective is to assess its potential in clinical diagnosis, prognosis, and as a future molecular target for therapy. The research findings reveal significant differences in HSPA4 expression across different cancer types, suggesting its diagnostic value and close association with cancer staging and patient survival rates. Furthermore, genetic variations and methylation status of HSPA4 play critical roles in tumorigenesis. Lastly, the interaction of HSPA4 with immune cells is linked to the tumor microenvironment (TME) and immunotherapy. In summary, HSPA4 emerges as a promising cancer biomarker and a vital member of the HSPs family, holding potential applications in diagnosis, prognosis, and immunotherapy.

## INTRODUCTION

The global incidence and mortality rates of cancer have emerged as a grave concern, with the worldwide burden of this affliction exhibiting an escalating trajectory [1]. Elucidating the mechanisms of cancer initiation and developing targeted therapeutic strategies hold significant importance. Historically, chaperone proteins have been posited to assist in the folding and refolding of proteins. When proteins exhibit defects or undergo irreversible misfolding, chaperones perform the dual function of error correction and degradation [2]. As a prominent subclass within the chaperone proteins, heat shock proteins (HSPs) have garnered significant attention in cancer research. Over the past few decades, the field of HSP research has witnessed substantial development and notable progress. Extensive researches on HSP inhibitors and vaccines have been conducted both domestically and internationally, demonstrating significant therapeutic effects [3–5]. Several studies indicate that HSPs play pivotal roles in several cancer-associated features, such as promoting tumor cell growth, supporting angiogenesis, and modulating tumor immune responses [6, 7]. Particularly noteworthy is the ongoing exploration of HSPs’ therapeutic potential in cancer treatment and immune modulation. Their emerging significance as targets for new drug development is increasingly recognized [8]. These research findings not only offer novel therapeutic strategies to researchers but also open new horizons for future medical studies.

HSPs can be classified into several families based on their apparent molecular weight and homology, including HSP90, HSP40, HSP60, and HSP70, among others [9]. HSP90 plays a role in signal transduction pathways alongside certain kinases and steroid receptors, and it may also serve as a “typical” molecular chaperone [10]. As a crucial co-chaperone protein, HSP40 is primarily involved in regulating the activity of HSP70 [11]. HSP60, with the assistance of ATP, facilitates the correct folding of 15-30% of cellular proteins [12]. HSP70 is considered to play a central role in maintaining protein homeostasis [13]. Its functions include preventing the adhesion of unfolded polypeptide chains, dissociating protein oligomers, participating in protein transport, and regulating the heat shock response. These members of the heat shock protein families play vital roles in organisms, primarily involving protein quality control and degradation. However, despite the overall functions of HSPs being well-established, there are still some areas where the specific functions of individual family members remain unclear. This article focuses on HSPA4, a key member of the HSP70 subfamily. HSPA4 is considered a type of “evil chaperone” within the HSPs family [14]. Recently, HSPA4 has come under the spotlight due to its potential oncogenic role across various cancers. Evidence suggests that HSPA4 is implicated in the pathogenesis and progression of hepatocellular carcinoma [15], colorectal cancer [16], gastric cancer [17], and head and neck cancer [14], positioning it as a potential therapeutic target and prognostic biomarker. While current research primarily focuses on the role of HSPA4 in specific cancers, its universal function across cancer types remains a relatively uncharted domain. Thus, a pan-cancer, in-depth study of HSPA4 is of paramount importance. Research into pan-cancer has unveiled the commonalities and specificities among different tumor lineages, thereby deepening our understanding of the mechanisms underlying tumor formation and progression [18]. In recent years, pan-cancer research integrating multiple omics analyses has been extensively utilized in the investigation of therapeutic targets and prognostic biomarkers [19, 20]. To delve deeper into the molecular mechanisms of tumorigenesis, we employed a myriad of bioinformatics approaches to investigate the role of HSPA4 in various cancers, aiming to assess its potential value in clinical diagnosis and prognosis. We also conducted a comprehensive analysis of HSPA4 from genetic variations, methylation, and immune-related perspectives. Our research seeks to elucidate the pivotal role of HSPA4, offering insights into its position within the HSPs family. We posit that HSPA4 stands as a robust biomarker for cancer, with applications in diagnosis and prognostic prediction, and may also emerge as a potential molecular target for future therapies.

## MATERIALS AND METHODS

### HSPA4 expression

The expression of the HSPA4 gene across a spectrum of cancer types has been meticulously studied utilizing the TIMER 2.0 database (http://timer.cistrome.org/). The full names and abbreviations of the cancers involved in this study are listed in Supplementary Table 1. A fold change greater than 2 was used as a criterion in this work. The expression of HSPA4 in pan-cancer was assessed to realize a comprehensive analysis by contrasting paired tumor and normal samples from the TCGA database. The analysis and visualization components of the study were conducted using R software (version 4.2.1), and the “ggplot2” R package was employed, placing pivotal emphasis on using the toiling process to harmonize the RNAseq data from TCGA [21]. To illustrate the disparity in expression between tumor tissues and their corresponding normal counterparts, the “Expression Analysis-Box Plot” module of the GEPIA2 (Gene Expression Profiling Interactive Analysis, version 2) platform (http://gepia2.cancer-pku.cn/#index) was leveraged to generate box plots, with data derived from the GTEx (Genotype-Tissue Expression) database [22]. Moreover, the protein expression levels of HSPA4 in various cancers were explored utilizing the Human Protein Atlas (HPA) database (images available from https://www.proteinatlas.org/ENSG00000170606-HSPA4/), and the subcellular localization of HSPA4 was ascertained through indirect immunofluorescence microscopy (images available from https://www.proteinatlas.org/ENSG00000170606-HSPA4/subcellular#human). The investigation was extended by engaging the CPTAC analysis from the UALCAN portal (dates available from https://ualcan.path.uab.edu/analysis.html) to assess the expression levels of HSPA4 protein across a variety of tumors, providing more detailed insights into its expression across different cancer entities.

### ROC curve and survival analysis

Employing the receiver operating characteristic (ROC) curve, an in-depth analysis of HSPA4 in common cancers was conducted. The construction of the ROC curve is reliant on the expression data of HSPA4 mRNA in tumors and their corresponding normal tissues, sourced from the TCGA and GTEx databases. Through the GEPIA2 tool, a correlation between HSPA4 expression in all TCGA tumors and patients’ pathological staging was investigated. Moreover, the Kaplan-Meier (K-M) method was utilized for the survival analysis of HSPA4, comparing the overall survival (OS), disease-specific survival (DSS), and progression-free interval (PFI) between high and low expression groups of HSPA4 [23]. The p-value in survival analysis was calculated using Cox regression analysis. All pertinent data and charts, including survival plots and forest plots, were analyzed and illustrated via the TaoXian Academic Online website (https://www.xiantaozi.com/).

### Genetic alteration analysis

Collect variant frequency, quantity of mutations, types of mutations, locations of mutations, and three-dimensional (3D) structural information of the HSPA4 gene across all TCGA tumors, utilizing the cBioPortal tool (https://www.cbioportal.org/). The UALCAN tool was applied to analyze the methylation levels of HSPA4 in normal and primary tumor tissues.

### Immune-related analysis

To assess the correlation between HSPA4 expression and immune infiltration levels in TCGA tumor tissues, a variety of algorithms, including MCPCOUNTER, TIDE, EPIC, XCELL, and TIMER, were utilized, and analyses were facilitated using the TIMER2.0 tool. To delve into the potential association of HSPA4 expression with molecular or immune subtypes in prevalent cancers, the “Subtype” module of the TISIDB database was employed. The relationship of HSPA4 expression to immunity was probed using the R “GSVA” package, deploying single-sample Gene Set Enrichment (ssGSEA) to elucidate the association of HSPA4 with 22 immune-related cells. The TISIDB database integrates multiple data types, providing a comprehensive view of the interplay between cancer and the immune system.

### Single-cell analysis

CancerSEA, distinguished as a specialized single-cell sequencing database, serves as a critical analytical tool for researching the functions of cancer cells at the single-cell level [24]. This database compiles 14 tumor-related cellular functions from 900 cancer cells originating from the United States, showcasing various functional states of cancer cells at the single-cell dimension. By examining data based on single-cell sequencing, correlations between HSPA4 expression and multiple tumor functions can be observed. The T-SNE plot further delineates the expression spectrum of HSPA4 in single cells within TCGA samples.

### GSEA

Utilizing Gene Set Enrichment Analysis (GSEA), differences in biological pathways between high and low HSPA4 groups can be discerned. The Xiantao Academic platform (https://www.xiantaozi.com/) offers an intuitive method for the visualization of enrichment results.

### Functional enrichment analysis

To integrate and analyze proteins potentially interacting with HSPA4, the protein-protein interaction (PPI) network from the HSPA4 STRING database (https://string-db.org/) was utilized. A confidence score and significance threshold were set at 0.7 to acquire related genes for PPI network analysis. Subsequently, the acquired data were imported into Cytoscape (Version 3.9) for visualization and further analysis. Through the utilization of the cytoHubba plugin in Cytoscape, key modules within the network were identified, and nodes ranked in the top 10 by MCC were denoted as hub genes. The relevance of these hub genes was also explored across various types of cancer, leading to the determination of proteins binding with HSPA4. Furthermore, the “Similar Gene Detection” feature of GEPIA2 was employed to identify the top 100 target genes related to snd1, based on datasets from all TCGA tumors and normal tissues. The interactive Venn diagram viewer, Jvenn [25], was used for cross-analysis to compare HSPA4-binding genes with interacting genes. In addition, a Gene Ontology (GO) functional and Kyoto Encyclopedia of Genes and Genomes (KEGG) enrichment analysis was performed on genes closely interacting with HSPA4, as obtained from the STRING database. This analysis was conducted via the Functional Clustering module [GOKEGG] in TaoXian Academic (https://www.xiantaozi.com/) and was visualized using bubble charts.

## RESULTS

### HSPA4 expression in pan-cancer

**Figure 1:**
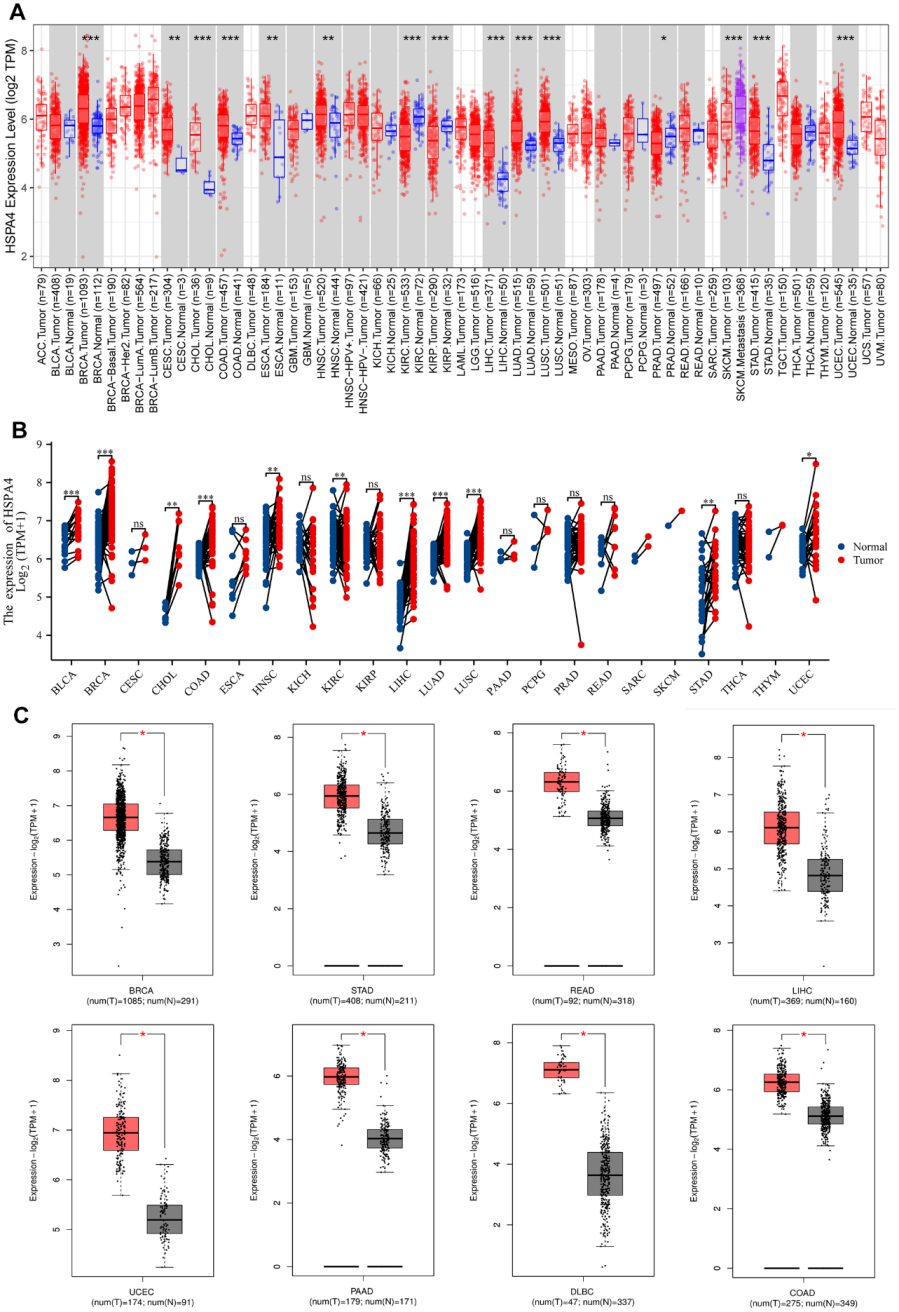
**Differential expression analysis of HSPA4.** (**A**) Expression of HSPA4 mRNA in pan-cancer. (**B**) The expression differences of HSPA4 in tumor and corresponding adjacent tissues were compared with paired analysis. Mann-Whitney U test was used for this analysis, ns, p≥0.05; *p< 0.05; **p<0.01; ***p<0.001. (**C**) Differential expression analysis of HSPA4 in BRCA, STAD, READ, LIHC, UCEC, PAAD, DLBC and COAD.

Across a broad spectrum of cancers, we assessed the expression levels of HSPA4 using TIMER2.0. As presented in Figure 1A, HSPA4 showcased a ubiquitous upregulation in tumors compared to their adjacent normal tissues. Specifically, elevated expression of HSPA4 was observed in BRCA, CESC, CHOL, COAD, ESCA, HNSC, LIHC, LUAD, LUSC, STAD, and UCEC, with the differences being statistically significant. Conversely, reduced expression of HSPA4 was identified in KIRC, KIRP, and PRAD when compared to normal tissues.

**Figure 2:**
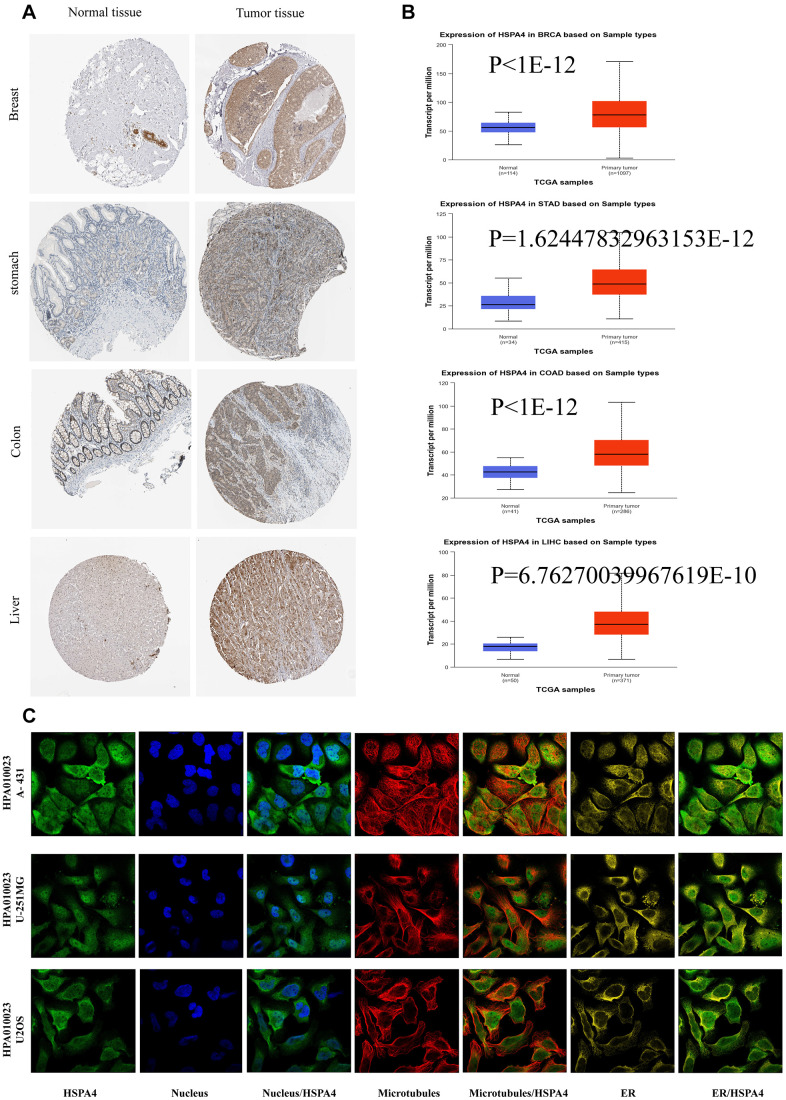
**The expression of HSPA4 protein in different cancers.** (**A**) Based on the HPA database (http://www.proteinatlas.org/), the expression of HSPA4 protein in normal or tumor tissue of breast, stomach, colon, liver, and thyroid was displayed (images available from https://www.proteinatlas.org/ENSG00000170606-HSPA4/). (**B**) Based on the UALCAN database, the expression of HSPA4 protein in normal or tumor tissue of breast, stomach, colon, liver, and thyroid was displayed (dates available from https://ualcan.path.uab.edu/analysis.html). (**C**) Immunofluorescence staining of the subcellular localization of HSPA4 in HPA database (images available from https://www.proteinatlas.org/ENSG00000170606-HSPA4/subcellular#human).

Further corroborations from TCGA and GTEx databases supported these findings (Figure 1B). Notably, increased HSPA4 expression was validated in BRCA, STAD, READ, LIHC, LUSC, DLBC, and COAD in comparison to their normal counterparts (Figure 1C). Using the UALCAN dataset from the National Cancer Institute, we assessed the protein expression levels of HSPA4 (Figure 2B). In conjunction with insights from the HPA database, we observed a significant overexpression of HSPA4 in cancerous tissues compared to normal tissues, particularly in the breast, stomach, colon, and liver (Figure 2A). In a comprehensive immunofluorescence localization study anchored on the HPA database, we meticulously analyzed the A-431, U-251 MG, and U2OS cells to decipher the subcellular localization of the HSPA4 protein. Through specific labeling of the nuclei, microtubules, and endoplasmic reticulum, our findings underscored a pronounced enrichment of HSPA4 within the microtubules and cytoplasm (Figure 2C). In summation, HSPA4 displays widespread elevated expression in a vast majority of cancers, notably those associated with the digestive system.

### Diagnostic value and prognostic value of HSPA4 in pan-cancer

**Figure 3:**
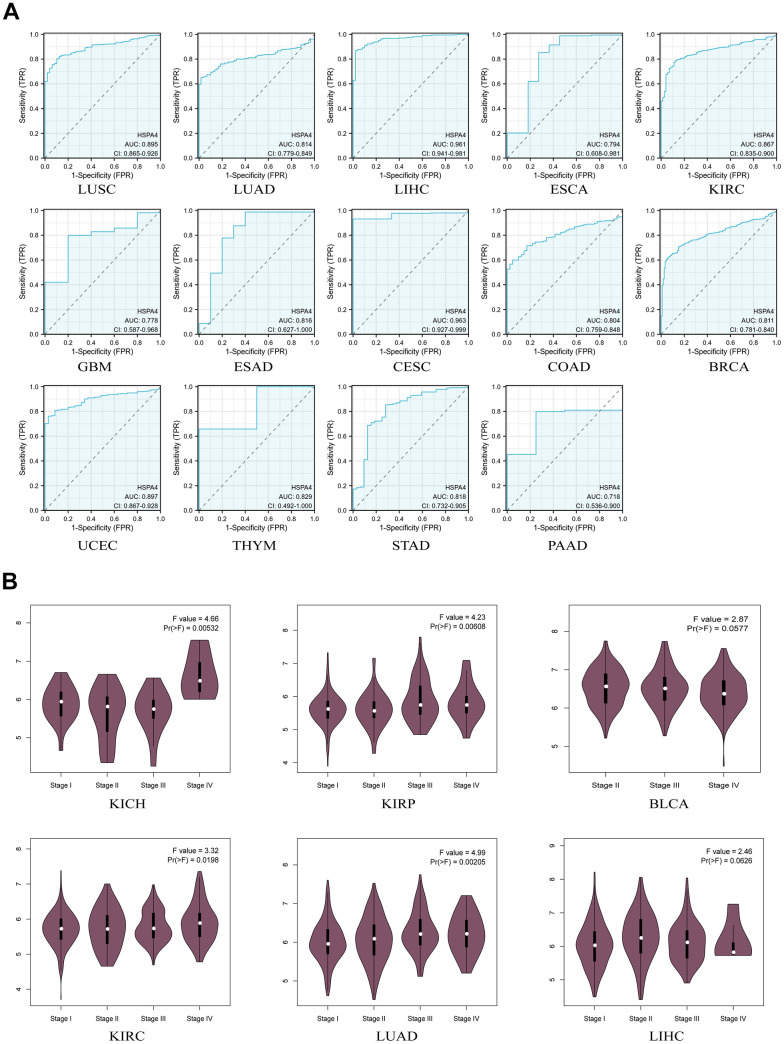
**Diagnostic value of HSPA4 in pan-cancer.** (**A**) ROC curve of HSPA4 in 14 types of cancers. (**B**) Based on the TCGA data, the expression levels of the HSPA4 gene were analyzed by the main pathological stages (stage I, stage II, stage III, and stage IV) of KICH, KIRP, BLCA, KIRC, LUAD, LIHC. Log2 (TPM+1) was applied for log-scale.

Although HSPA4 has been observed to exhibit elevated expression levels in tumor tissues compared to normal tissues, its potential utility in cancer diagnosis and prognosis remains to be elucidated through further in-depth studies. To evaluate the diagnostic significance of HSPA4 across a spectrum of cancers, we employed the ROC curve analysis. An area under the curve (AUC) value greater than 0.7 was considered to indicate high accuracy [26]. HSPA4 demonstrated AUC values exceeding 0.7 in the ROC analyses for LUSC, LUAD, LIHC, ESCA, KIRC, GBM, ESAD, CESC, COAD, BRCA, UCEC, THYM, STAD, and PAAD (Figure 3A). Furthermore, analysis from CPTAC revealed a correlation between HSPA4 expression and the pathological staging of cancers such as KICH, KIRP, BLCA, KIRC, LUAD, and LIHC, suggesting a potential influence of HSPA4 on the staging of these malignancies (Figure 3B). This underscores the potential diagnostic value of HSPA4 in these malignancies.

**Figure 4:**
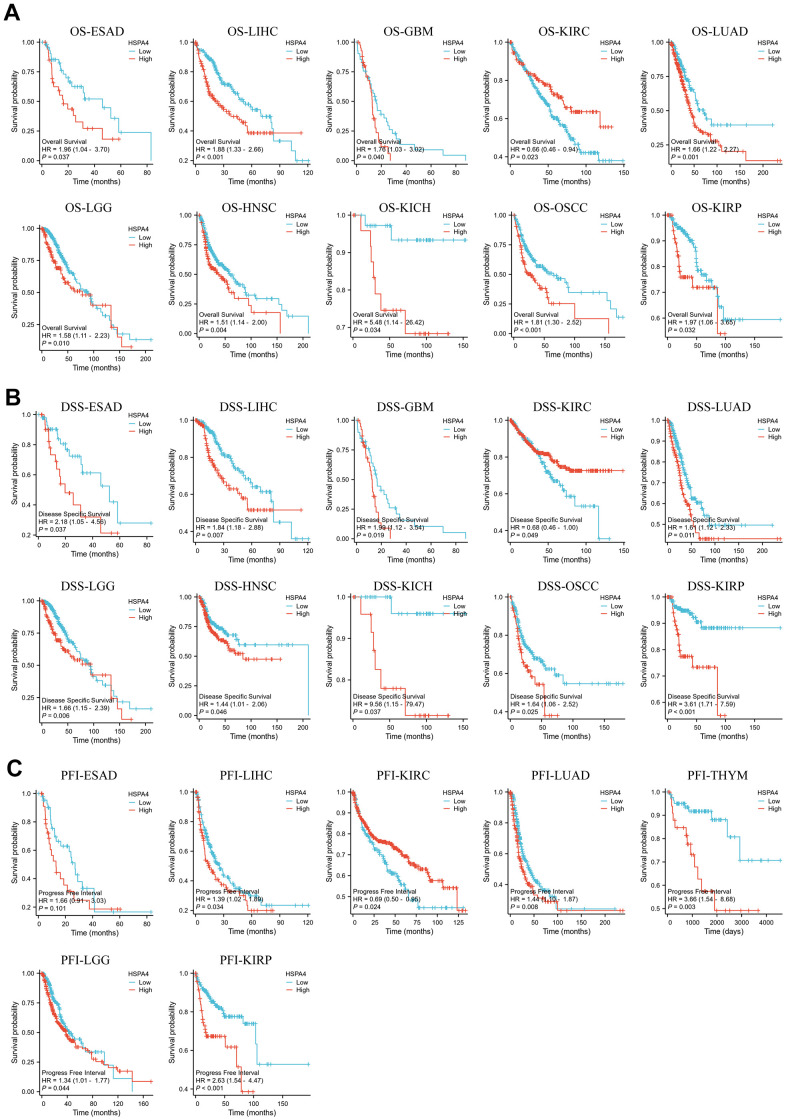
**Survival prognosis analysis of cancers.** (**A**) OS analysis of HSPA4 gene in the TCGA dataset. (**B**) DSS analysis of HSPA4 gene in TCGA database. (**C**) PFI analysis of HSPA4 gene in TCGA database.

We further stratified the cancer patients into high and low HSPA4 expression groups and utilized the TCGA dataset to investigate the association between HSPA4 expression and patient prognosis. Using Kaplan-Meier survival plots combined with univariate Cox regression analysis, we assessed the prognostic relevance of HSPA4 in diverse cancers (Figure 4A). Intriguingly, elevated HSPA4 expression correlated with diminished survival rates in patients with ESAD, LIHC, GBM, LUAD, LGG, HNSC, KICH, OSCC, and KIRP. Conversely, in KIRC patients, a surge in HSPA4 expression was linked to an extended survival duration (P= 0.023). Moreover, additional validation on DSS showcased a trend aligned with OS outcomes (Figure 4B). In ESAD, LIHC, LUAD, THYM, LGG, and KIRP, high HSPA4 expression significantly associated with a detrimental PFI (Figure 4C). Employing univariate Cox regression analysis, we scrutinized the association between HSPA4 expression and OS, DSS, and PFI across different cancer types. The forest plot exhibited results resonating with the previous findings (Supplementary Figure 1). Collectively, heightened HSPA4 expression implies diminished survival rates in a majority of cancer types. Our findings illuminate the intrinsic association between HSPA4 expression and the diagnosis and prognosis of various cancers, underscoring its potential clinical relevance.

### HSPA4 gene mutation and promoter methylation level in pan-cancer

**Figure 5:**
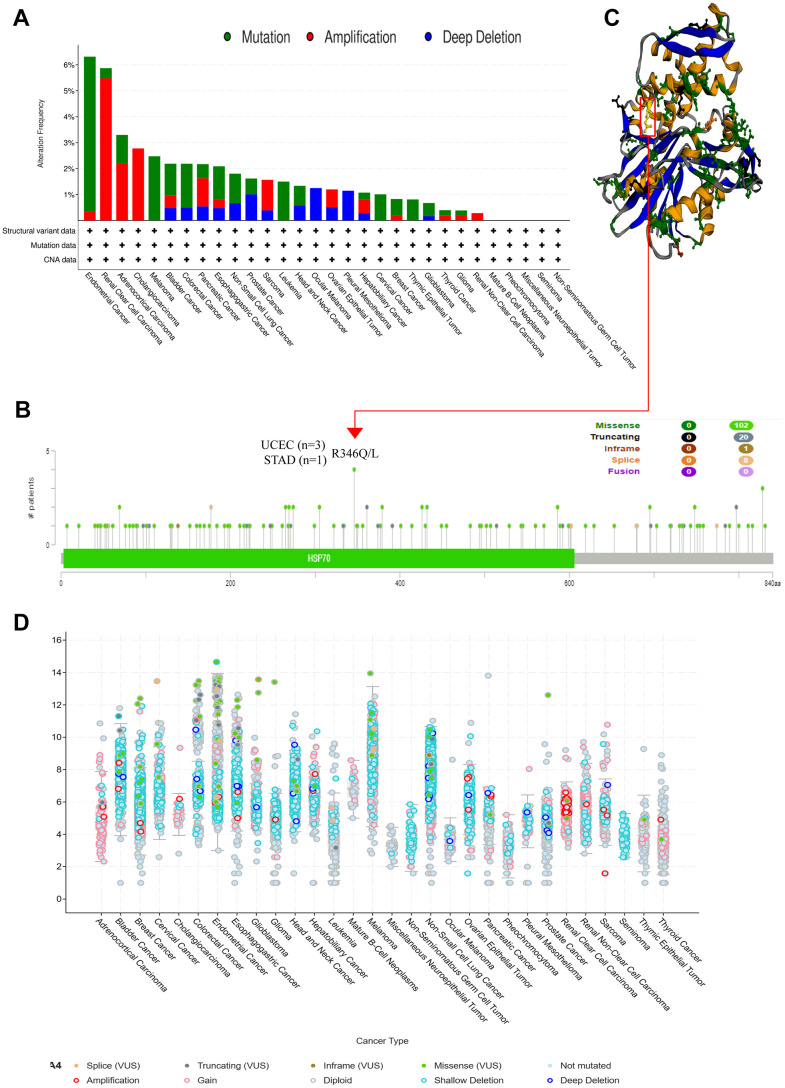
**HSPA4 gene mutation and promoter methylation level in pan-cancer.** cBioPortal was used to show the frequency of variation in different mutation types (**A**) and mutation sites (**B**) of HSPA4 in pan-cancer. (**C**) R346Q/L mutation site in the 3D protein structure of HSPA4. (**D**) Mutation counts and types of HSPA4 in 30 cancers.

To comprehensively elucidate the genetic alterations of HSPA4 in various cancers, we employed the cBioPortal tool, leveraging the TCGA database, to meticulously investigate its genetic mutations across numerous tumor samples. Remarkably, within endometrial cancer samples, we discerned a conspicuous mutation frequency in HSPA4 exceeding 6%. Of these, over 5% were classified as “mutation” type alterations (Figure 5A). We offer a granular view of the types, loci, and number of cases affected by HSPA4 gene alterations (Figure 5B). Notably, missense mutations emerged as the predominant mutation pattern within HSPA4. Mutations at the R346Q/L locus were detected in three UCEC cases and one STAD case. Figure 5C further delineates the structural ramifications of these R346Q/L alterations in the 3D conformation of the HSPA4 protein. In a survey of 30 distinct cancer types, the majority exhibit a shallow deletion of HSPA4 mRNA (Figure 5D). Intriguingly, amplification of HSPA4 mRNA is notably prevalent in KIRC.

Moreover, the methylation status of the HSPA4 promoter region and its implications for diverse cancers remained central to people’s inquiries. Existing studies affirm that methylation levels within the promoter region can modulate gene transcription, subsequently influencing tumor genesis and progression [27]. Hence, elucidating the methylation patterns of HSPA4 in cancers is pivotal for understanding its role in tumorigenesis and progression. Compared to normal tissue counterparts, we observed elevated methylation levels of HSPA4 in cancer types, including LIHC, CHOL, THCA, HNSC, KIRP, LUSC, and BRCA. Contrarily, in COAD and READ cancers, a significant reduction in HSPA4 promoter methylation was discerned (Supplementary Figure 2A–2G). Such findings intimate that transcriptional expression of HSPA4 may be modulated by alterations in its promoter methylation. In conclusion, the genetic variations and methylation patterns of HSPA4 across various cancers furnish novel insights, potentially expounding its function and role in oncology.

### Immune-related analysis

### Immune infiltration analysis

The intricate landscape of the tumor microenvironment (TME) has recently emerged as a focal point of cancer research. Within this milieu, immune cell infiltration stands out, with mounting evidence associating it with cancer onset, progression, and metastasis [28, 29]. Notably, cancer-associated fibroblasts (CAFs) are recognized for modulating the functions of a plethora of infiltrating immune cells, impacting both directly and indirectly the viability and activities of tumor cells [30, 31].

Harnessing the computational power of the EPIC, MCPCOUNTER, XCELL, and TIDE algorithms, we embarked on an exhaustive assessment of the dynamic relationship between CAFs infiltration and HSPA4 gene expression across diverse tumor types in the TCGA database. Our data revealed a pronounced positive correlation in CESC, HNSC, and PAAD. Contrarily, TGCT displayed a negative association (Supplementary Figure 3A). Leveraging advanced statistical techniques, scatter plots were generated to visually depict the correlation between HSPA4 expression and CAFs infiltration across the mentioned tumors (Supplementary Figure 3B). This study furnishes a novel perspective on the nexus between immune cell infiltration and pivotal gene expression within the TME, holding promise to inform and refine therapeutic strategies in oncology.

### Analysis of HSPA4 and immune cells and immune subtyping

**Figure 6:**
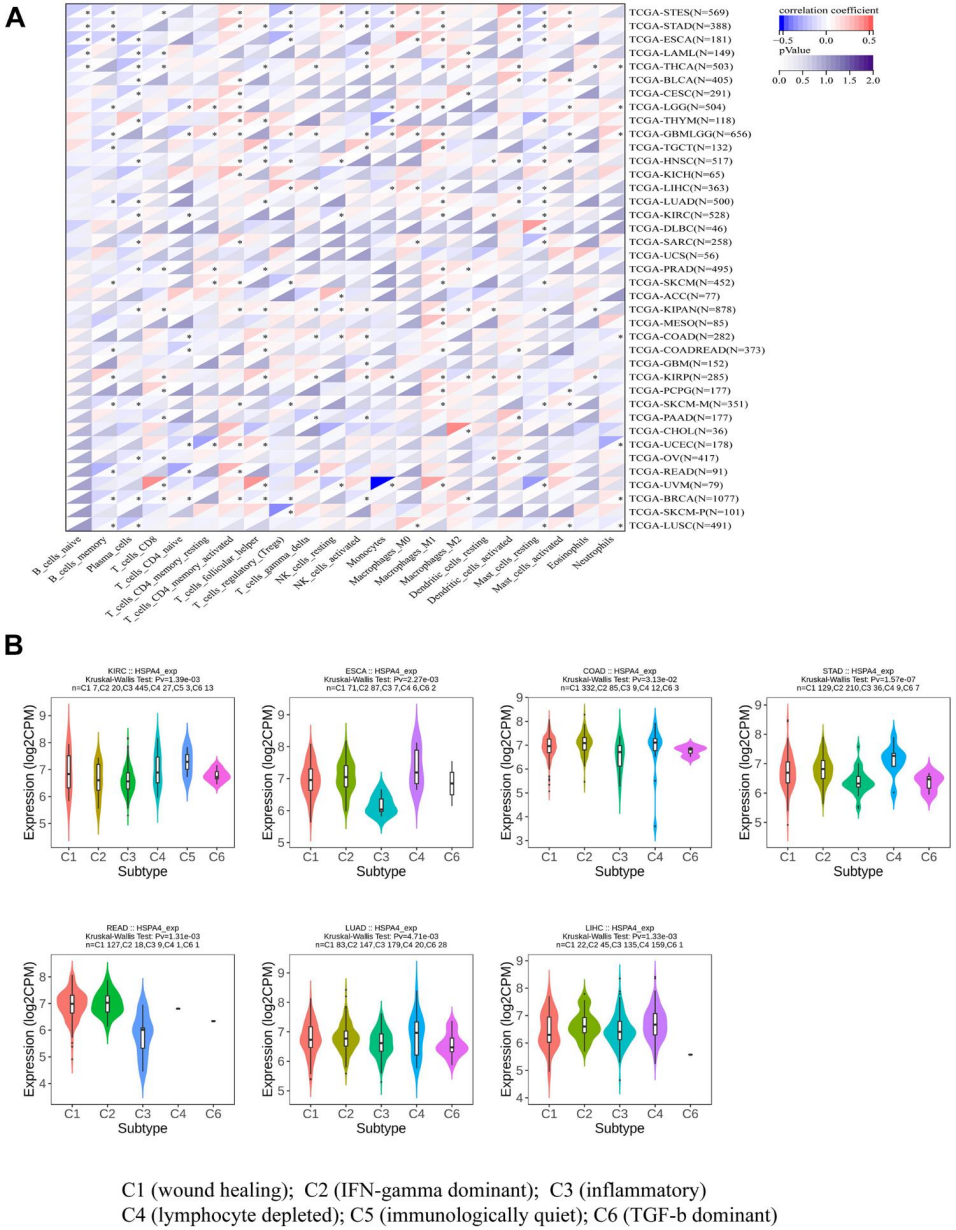
**HSPA4 in relation to immune cell and immune subtyping analysis.** (**A**) Association analysis between pan-cancer and immune-related cells. (**B**) Correlation between HSPA4 expression and immune subtypes in seven cancers.

To gain deeper insights into the direct link between immune cells and HSPA4, we utilized a specific methodology, employing immune-associated cells to generate a heatmap depicting the expression of HSPA4 (Figure 6A). This analytical approach revealed varying degrees of association between HSPA4 and diverse tumors and immune cells. Furthering our investigation, we assessed the expression patterns of HSPA4 within immune subtypes across seven distinct cancers. Our results highlighted marked discrepancies in HSPA4 expression amongst subtypes in cancers including KIRC (spanning 6 subtypes), ESCA (5 subtypes), COAD (5 subtypes), STAD (5 subtypes), READ (5 subtypes), LUAD (5 subtypes), and LIHC (5 subtypes) (Figure 6B). In essence, these observations accentuate the potential central role of HSPA4 in modulating immune responses and influencing cancer progression. Advanced research on HSPA4 not only holds the promise to elucidate its precise function in oncology but might also herald innovative avenues for future cancer therapeutic endeavors, particularly enhancing the efficacy of immunotherapeutic strategies.

### The expression pattern of HSPA4 at single-cell levels

**Figure 7:**
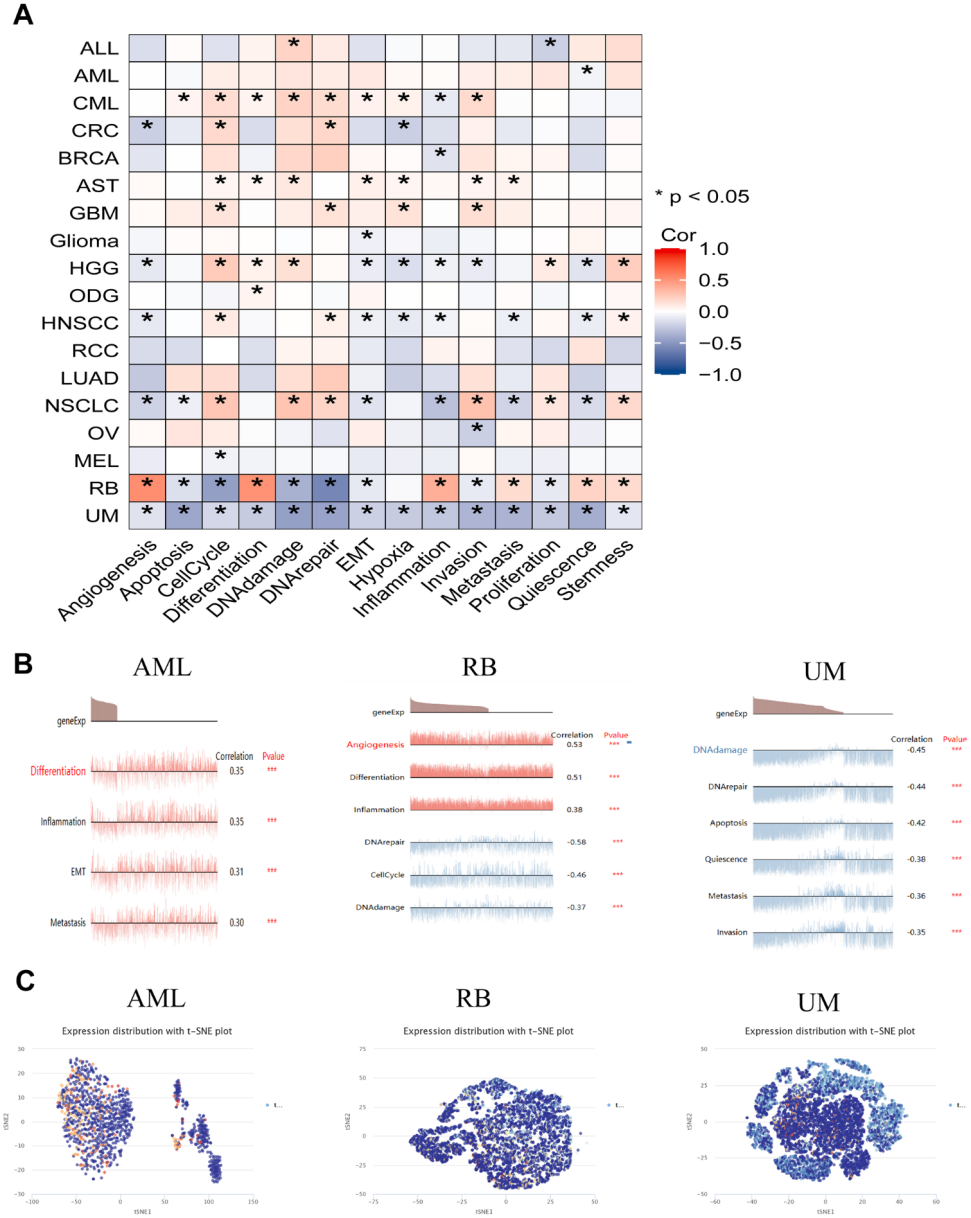
**The expression levels of HSPA4 at single-cell levels.** (**A**) Through the CancerSEA platform, we delved into the interplay between HSPA4 expression in tumors and its diverse functional states. (**B**) Functional landscape of HSPA4 in AML, RB, and UM. (**C**) Single-cell expression panorama of HSPA4 in AML, RB, and UM visualized via a T-SNE plot. * p < 0.05; ** p < 0.01; *** p < 0.001.

Single-cell transcriptomics serves as an efficacious approach, enabling in-depth exploration of candidate molecule functionalities at the cellular level. To delve deeper into the potential role of HSPA4 in tumors, we employed the CancerSEA tool to characterize the functional attributes of HSPA4 at the single-cell level. We observed that the expression of HSPA4 across various tumors exhibited certain conserved relationships with specific functional states while also revealing distinct associations (Figure 7A). These functions encompass angiogenesis, apoptosis, cell cycle, differentiation, DNA damage, DNA repair, epithelial-mesenchymal transition (EMT), hypoxia, inflammation, invasion, metastasis, proliferation, quiescence, and stemness. A particular emphasis was placed on the correlation of HSPA4 with functional states in specific tumor types (Figure 7B). In AML, HSPA4 expression demonstrated a positive correlation with differentiation, inflammation, EMT, and metastasis. In contrast, within RB, HSPA4 expression was positively correlated with angiogenesis, differentiation, and inflammation but was inversely related to DNA repair, cell cycle, and DNA damage processes. In UM, HSPA4 showcased a negative correlation with a range of biological behaviors, including DNA damage, DNA repair, apoptosis, quiescence, metastasis, and invasion. Furthermore, we illustrated the expression patterns of HSPA4 at the single-cell level in AML, RB, and UM using a T-SNE plot (Figure 7C).

### GSEA enrichment analysis

To delve deeper into the biological roles and significance of HSPA4 across diverse tumor tissues, we employed the GSEA approach, meticulously analyzing 12 distinct cancer types, namely ESAD, GBM, HNSC, KIRC, LIHC, LUAD, OSCC, PAAD, STAD, BRCA, CESC, and COAD (Supplementary Figure 4A–4L). Our findings underscore the pivotal role of HSPA4 in several key biological processes. HSPA4 was notably indispensable in cell cycle regulation, immune checkpoint pathways, B/T cell activation responses, interferon signaling transduction, and DNA replication, among others. These insights further emphasize the central position of HSPA4 in tumor biology. Understanding the functions of HSPA4 not only offers profound insights into its roles in tumor progression but may also pave the way for innovative therapeutic strategies and targets in the future.

### The PPI network and functional enrichment analysis of HSPA4

**Figure 8:**
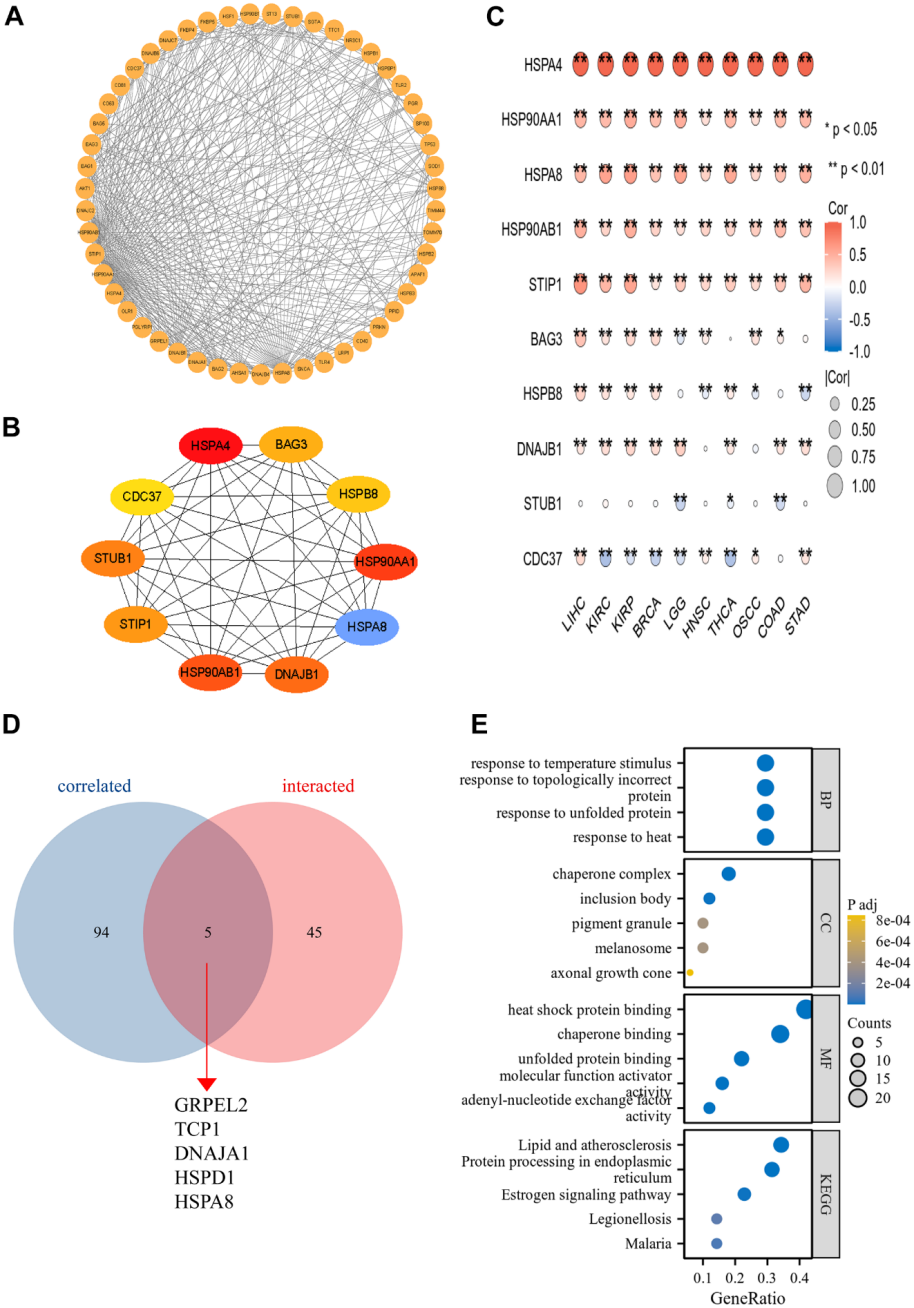
**The PPI network and functional enrichment analysis of HSPA4.** (**A**) The PPI network of HSPA4. (**B**) The top ten hub genes in the PPI network. (**C**) Heatmap of hub genes correlated with HSPA4 across ten cancers. *p < 0.05, **p < 0.01. (**D**) Cross-analysis was conducted between HSPA4-correlated genes and intersection genes. (**E**) GO/KEGG pathway enrichment for HSPA4 and its interacting genes.

Utilizing the STRING database, we retrieved genes closely associated with HSPA4 and subsequently constructed a PPI network (Figure 8A). Upon analysis, the top ten hub genes were identified as HSPA4, BAG3, HSPB8, HSP90AA1, HSPA8, DNAJB1, HSP90AB1, STIP1, STUB1, and CDC37 (Figure 8B). In cancers where HSPA4 expression significantly impacts prognosis, four genes—HSP90AA1, HSPA8, DNAJB1, and HSP90AB1—demonstrated a pronounced correlation (Figure 8C). A cross-comparative analysis between HSPA4-correlated genes and interacted genes revealed five overlapping genes: GRPEL2, TCP1, DNAJA1, HSPD1, and HSPA8 (Figure 8D). The proteins encoded by GRPEL2 and HSPD1 genes play pivotal roles in the mitochondria, aiding in the accurate folding and assembly of proteins. The protein encoded by the TCP1 gene is a key component of the molecular chaperone complex. In conjunction with other molecular chaperones, DNAJA1 protein participates in the protein folding process. HSPA8 is primarily involved in regulating cellular stress responses. These proteins are significantly associated with the functions of HSPs molecular chaperones and are of vital importance in cellular biology and physiological processes. Furthermore, using data sourced from the STRING database, we conducted GO/KEGG enrichment analysis (Figure 8E). The RNA functional categorizations encompassed biological processes (BP), molecular functions (MF), and cellular components (CC). The results predominantly indicated associations with characteristic heat shock protein functions, such as protein folding. Moreover, KEGG pathway analysis highlighted a significant association with the cell cycle.

## DISCUSSION

Recent advancements in clinical cancer screening and treatment face three main challenges: the lack of symptoms in early-stage cancers, the need for personalized treatment due to cancer’s heterogeneity, and the absence of effective biomarkers for certain cancers, complicating diagnosis and treatment monitoring. HSPs have garnered significant attention in tumor biology, especially with their pivotal role in tumorigenesis and cancer progression. The mechanisms of action of HSPs in cancer therapy primarily involve their roles in modulating the immune system and influencing the TME. HSPs can bind to antigenic peptides on the surface of cancer cells, forming HSP-peptide complexes, which facilitate the uptake, processing, and presentation of tumor-specific antigens, subsequently presented by antigen-presenting cells (APCs) to CD8+ cytotoxic T lymphocytes [32]. However, HSPs can also promote the growth, proliferation, and metastasis of tumor cells and mediate tumor cell resistance to stressors, contradicting their role in host protection [33]. Additionally, HSPs may induce T-cell tolerance, thereby creating an immunosuppressive environment conducive to cancer progression. Studies have shown that a variety of HSP inhibitors demonstrate potential therapeutic efficacy in cancer treatment, potentially by disrupting the ATPase activity of HSPs within cancer cells, thereby blocking oncogenic pathways and leading to apoptosis or loss of proliferative capacity in cancer cells [3, 4, 34, 35]. Moreover, cancer vaccines based on HSPs have been proven effective against a range of tumors expressing specific antigens, making them ideal targets for cancer vaccine therapy [5]. Current clinical trials targeting HSPs through inhibitors (NCT04437420) and vaccines (NCT03650257) are actively underway, primarily focusing on exploring the potential of novel anti-cancer therapies. However, the clinical application of HSP inhibitors faces several challenges, such as the induction of heat shock responses, retinal pathologies, and gastrointestinal toxicity [36, 37]. These challenges not only impose stringent safety requirements for patients but also present significant difficulties for researchers in further developing these inhibitors. On the other hand, research on vaccines, particularly their potential in cell-mediated immune activation, still encounters many unresolved issues [38]. The regulation of cell-mediated immune responses is crucial for vaccine efficacy. Delving into the role of HSPs family members within the TME and the immune system, and designing more precise targeted treatment strategies based on these insights, can provide pivotal understanding for the development of more effective immunotherapeutic methods.

Within the HSPs family, the member encoded by the HSPA4 gene stands out. This gene demonstrates a profound association with various cancer types. In non-small cell lung cancer, HSPA4 has shown its potential as an oncogene [39]. Inhibition of the HSPA4 gene substantially diminishes the invasive and metastatic capabilities of tumor cells [39]. Similarly, in head and neck squamous cell carcinoma, HSPA4 not only emerges as a promising therapeutic target but also might serve as a prognostic biomarker [14]. In breast cancer patients, pathogenic IgG targeting glycosylated HSPA4 is produced in sentinel lymph nodes by B cells [40], potentially accelerating lymph node metastasis. The relationship between HSPA4 and hepatocellular carcinoma has also been scrutinized. Bioinformatic studies highlight that high HSPA4 expression is significantly correlated with poor hepatocellular carcinoma prognosis, underscoring its central role in immune modulation [41]. Intriguingly, the progression of colorectal cancer can be effectively curbed by decreasing HSPA4 expression [16]. In gastric cancer, specific mutations related to HSPA4 are significantly associated with tumorigenesis [17]. Despite a multitude of studies examining HSPA4’s role in specific cancer types, a holistic perspective remains lacking. Addressing this gap, we embarked on the first comprehensive investigation of HSPA4 across cancers, aiming to elucidate its broader biological significance.

Delving deep into the TIMER2.0 tool, TCGA, GTEx, and CPTAC databases, we found marked variability in HSPA4 expression across multiple cancers. Notably, bioinformatics analyses reveal that HSPA4 is significantly overexpressed in gastric, colorectal, and liver cancers. Previous studies also indicate that HSPA4 plays a pivotal role in these gastrointestinal tumors [16, 17, 41]. Furthermore, we hypothesize that the link between HSPA4 and gastrointestinal tumors might be closely related to changes in the TME’s stress response, including reduced glucose and oxygen availability and environmental acidification [42, 43]. However, the precise molecular mechanisms underlying the association between gastrointestinal tumors and HSPA4 require further investigation to elucidate. This suggests HSPA4’s crucial role in cancer progression. Conversely, its expression in KIRC and PRAD was significantly diminished. Such variation unveils the intimate relationship between HSPA4, cellular specificity, and underlying BP. Further investigations into HSPA4’s localization revealed significant enrichment in microtubules and cytoplasm. Given that microtubules play an essential role in maintaining cell morphology, material transport, and cell division, their intimate association with oncogenesis becomes evident [44]. Furthermore, our ROC analysis demonstrated a noteworthy diagnostic value of HSPA4 across various cancers, with its AUC frequently surpassing the 0.7 mark, strongly indicating its potential as a bio-marker for oncology. Delving deeper into the CPTAC dataset, a pronounced correlation was discerned between HSPA4 expression and the pathological staging of cancer. To understand this nexus with greater precision, we stratified based on HSPA4 expression levels and meticulously examined its association with patients’ OS, DSS, and PFI. OS is a widely-utilized metric primarily employed to assess the impact of treatment on the overall extension of a patient’s life. It encompasses all causes of death, thereby providing a comprehensive measure of survival. DSS focuses on deaths directly attributable to the disease itself, more accurately reflecting the mortality risk associated with the specific illness. On the other hand, PFI centers on evaluating the efficacy of treatments in delaying disease progression. Intriguingly, in our analysis, HSPA4 demonstrated correlations with these survival indices across multiple tumor types, typically exhibiting consistent trends in most cases. In the majority of cancers, an elevated expression of HSPA4 was concomitant with a diminished survival rate. However, distinctively in KIRC, heightened HSPA4 expression seemed to serve as a favorable prognostic indicator for extended survival. Collectively, augmented expression of HSPA4 is generally linked with an adverse cancer prognosis, especially pronounced in cancers like ESAD, LIHC, and LUAD. Given the paramount diagnostic and predictive prowess HSPA4 showcases as a cancer biomarker, it’s evident that while its high expression commonly suggests an unfavorable outcome, exceptions like KIRC do exist. This underscores the intricacies inherent in cancer’s pathogenesis and progression, emphasizing the need for an in-depth exploration of specific tumor types even within the broader scope of oncological research.

Upon rigorous exploration of genetic variations in the HSPA4 gene across diverse cancers, we identified significant mutations of HSPA4 in several tumor types. Specifically, in UCEC, the mutation frequency of HSPA4 notably surpassed 6%, with over 5% of these categorically classified as “mutations”. Missense mutations can induce significant alterations in protein structure and function, conferring a selective growth advantage to cancer cells [45]. Importantly, missense mutations dominated the mutation spectrum of HSPA4. From an analysis spanning 30 distinct cancer types, we detected a prevalent shallow deletion in HSPA4 mRNA across the majority of tumors. Strikingly, in KIRC, an extraordinary amplification of HSPA4 mRNA was observed, potentially playing a critical regulatory role in cancer progression. Moreover, the methylation status of the HSPA4 promoter region plays a pivotal role in tumorigenesis, progression, and its prognostic implications for patients [41]. Our data revealed that, relative to their normal tissue counterparts, there was a pronounced increase in the methylation levels of the HSPA4 promoter region in various tumor types. Conversely, in COAD and READ, a significant reduction in its methylation was identified. These findings suggest that the transcriptional activity of HSPA4 may be modulated by the methylation status of its promoter region. Collectively, our insights into the genetic variations and methylation patterns of HSPA4 across different cancers provide a novel perspective on its functional role in oncology, laying the groundwork for potential targeted therapeutic strategies in the future.

In recent years, the TME and the infiltration of immune cells therein have emerged as focal points in cancer research, a trend substantiated by numerous studies [28, 29, 46]. Notably, CAFs are believed to regulate the function of infiltrating immune cells [30, 31]. Our research indicates a pronounced positive correlation between HSPA4 gene expression and CAFs infiltration in CESC, HNSC, and PAAD, while a negative correlation was observed with TGCT. B cells and T cells are central functional players in the immune response. B cells possess the capability to produce a diverse set of antibodies, thereby playing an instrumental role in mediating the immune response. When the function and quantity of immune cells are compromised, tumor cells may exploit this vulnerability to evade immunosurveillance, posing a detrimental impact on the OS of cancer patients [47]. In our heatmap analysis focused on HSPA4 expression with respect to immune cells, a significant correlation between B cells, T cells, and various cancers emerged, suggesting their integral role in the oncogenic progression modulated by HSPA4. Further investigations revealed distinct expression patterns of HSPA4 across immune subtypes in KIRC, ESCA, COAD, STAD, READ, LUAD, and LIHC. These findings underscore the pivotal role of HSPA4 in modulating immune responses, cancer progression, and immune subtypes. Over recent years, immunotherapy has increasingly emerged as the focal point of research in the realm of cancer treatment, demonstrating remarkable efficacy in various tumor therapies [48]. A deeper understanding of HSPA4 not only unveils its central function in oncology but also offers potential avenues for cancer treatment, especially in refining immunotherapeutic strategies [49].

In our exploration of the functional role of HSPA4 in tumorigenesis, we employed single-cell transcriptomics and the CancerSEA tool. CancerSEA serves as a unique platform for delving into the functional states of cancer cells at the single-cell level, providing invaluable insights for cancer research and gene-function association analyses [24]. Our findings indicate a significant association between HSPA4 and various functional states across different tumors, with its role in cell cycle regulation being particularly pronounced. It is well-understood that tumorigenesis is closely linked to aberrant cell proliferation, a process tightly governed by the cell cycle control mechanism [50]. However, in cancer cells, the failure of damage checkpoints allows these cells to continue dividing despite accumulating genetic defects [51]. Intriguingly, previous studies have shown that knocking down HSPA4 results in a G2 phase arrest, suggesting a novel therapeutic avenue for colorectal cancer [16]. Through GSEA enrichment analysis, we further discerned that HSPA4 plays a pivotal role in various tumors, especially in pathways related to cell cycle regulation, immune checkpoint pathways, and activation responses of B/T cells. These novel findings furnish invaluable insights into the role of HSPA4 in the landscape of cancer single-cell studies.

In the present study, we harnessed the STRING database to explore genes associated with HSPA4, culminating in the successful construction of a PPI network. Our meticulous analysis identified ten core genes intricately linked with HSPA4. Crucially, upon evaluating genes that may significantly influence cancer prognosis, HSP90AA1, HSPA8, DNAJB1, and HSP90AB1 manifested pronounced correlations with HSPA4 expression. A cross-comparative analysis of genes related to HSPA4 and their interacting counterparts spotlighted five overlapping genes: GRPEL2, TCP1, DNAJA1, HSPD1, and HSPA8. Delving deeper using data from the STRING database, we embarked on a GO and KEGG enrichment analysis, aiming for a profound understanding of these genes’ functions and interplay within the cellular milieu. Our GO categorization spanned BP, MF, and CC, granting us insights into the gene’s functionality and prospective cellular localization [52]. It is noteworthy that these genes were intimately tied to the quintessential functions of the HSPs family, particularly protein folding. Protein folding, an essential BP within the cell, ensures proteins achieve their correct 3D conformations, thereby facilitating their designated roles. Despite the considerable advances in heat shock protein research, the specific role and mechanism of HSPA4 within this family warrant further elucidation. Furthermore, our KEGG pathway analysis underscored a tight association of these genes with cell cycle regulation, resonating with our earlier single-cell analysis outcomes. This paves the way for future investigations into the potential roles of these genes in cancer and other diseases.

In summary, we have conducted a comprehensive pan-cancer analysis of HSPA4 for the first time. Across various cancer types, the HSPA4 gene emerges as a pivotal player, underscoring its central role in tumorigenesis and cancer progression. Notably, HSPA4 not only shows potential as a key biomarker for malignancies, but its elevated expression is also frequently associated with poorer patient outcomes. Upon deeper investigation, genetic variations of HSPA4, specific methylation patterns, and its interaction with immune cells all appear to be critical determinants of its function in tumors. Moreover, HSPA4 plays a crucial role in maintaining protein homeostasis, which is vital for cell function and survival. This gene is closely intertwined with essential biological processes such as the cell cycle, immune responses, and protein folding. Elucidating these associated genes will empower us to devise more precise and targeted cancer therapeutic strategies. Based on these findings, there is reason to propose that HSPA4 could serve as a promising biomarker for the diagnosis and prognosis of various types of cancer, and as a potential target for immunotherapy. Although significant progress has been made in understanding HSPA4, there remain some limitations. Previous studies have largely focused on individual cancer types, lacking a comprehensive understanding of HSPA4’s overall role. Moreover, our research has primarily been based on database analysis, with a shortage of validation using a broader range of clinical samples. Given the complexity of cancer, HSPA4 may play varying roles in different cancer types, necessitating further research for deeper exploration. Future studies should emphasize conducting more clinical research to verify the role of HSPA4 in human cancers and its potential as a therapeutic target. Understanding how HSPA4 interacts with other proteins and signaling pathways, and how these interactions impact cancer development and treatment, are also crucial. Exploring the application of HSPA4 inhibitors or vaccines in cancer therapy is another important research direction. In summary, despite certain progress, many unknown areas in HSPA4 research remain to be explored further, to better harness this protein’s potential in cancer therapy.

## Supplementary Material

Supplementary Figures

Supplementary Table 1
