# Supplementary material for: Unveiling the potential of HSPA4: a comprehensive pan-cancer analysis of HSPA4 in diagnosis, prognosis, and immunotherapy
Source: Aging (Albany NY). 2024 Jan 31;16(3):2517–41. doi: 10.18632/aging.205496 (PMC10911360; doi:10.18632/aging.205496)
Supplement: Supplementary Figures [file aging-16-205496-s001.pdf]

SUPPLEMENTARY FIGURES

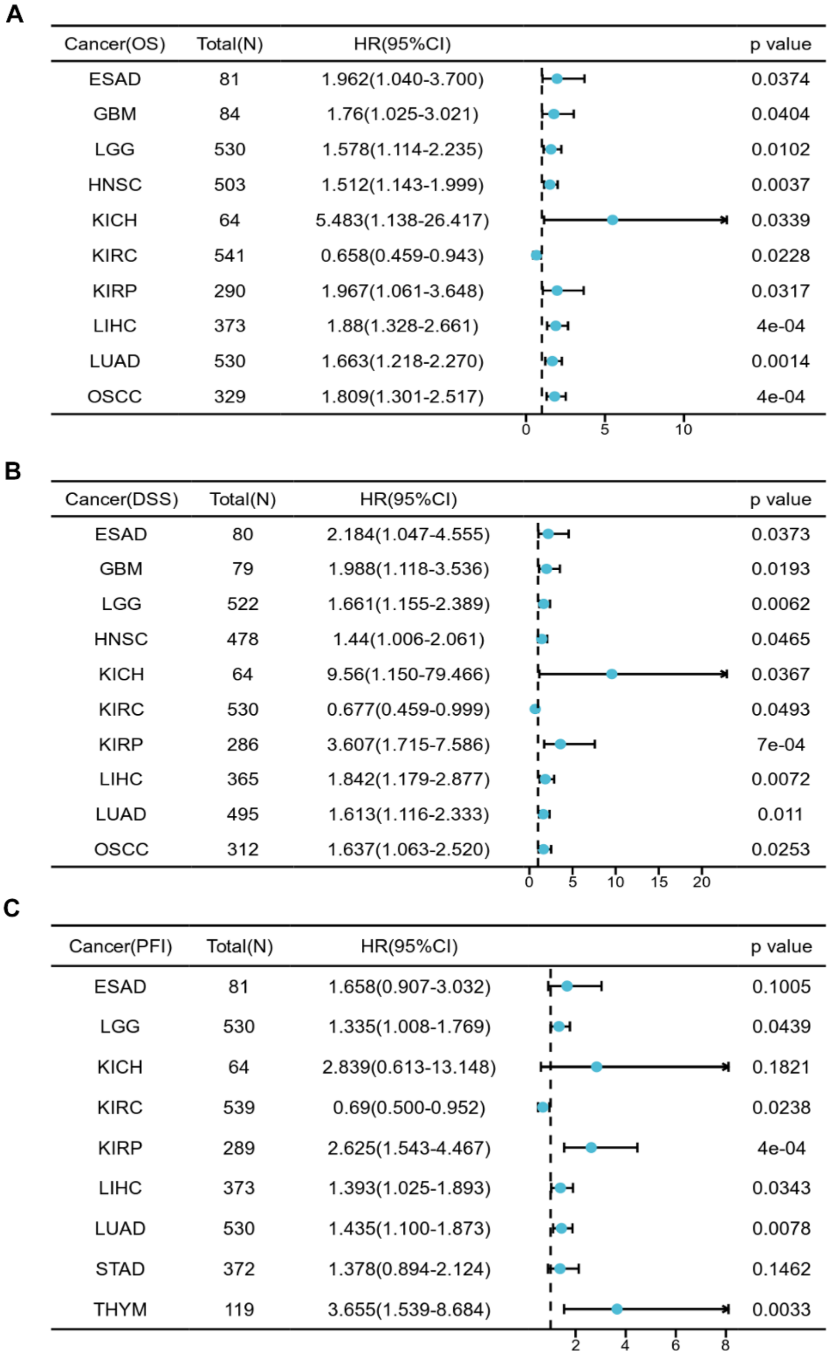

Supplementary Figure 1. Survival prognosis analysis of cancers. (**A**) Illustrated is a forest plot delineating the association between the HSPA4 gene and Overall Survival (OS) in the TCGA dataset. (**B**) Illustrated is a forest plot delineating the association between the HSPA4 gene and Disease-Specific Survival (DSS) in the TCGA database. (**C**) Illustrated is a forest plot delineating the association between the HSPA4 gene and Progression-Free Interval (PFI) in the TCGA database.

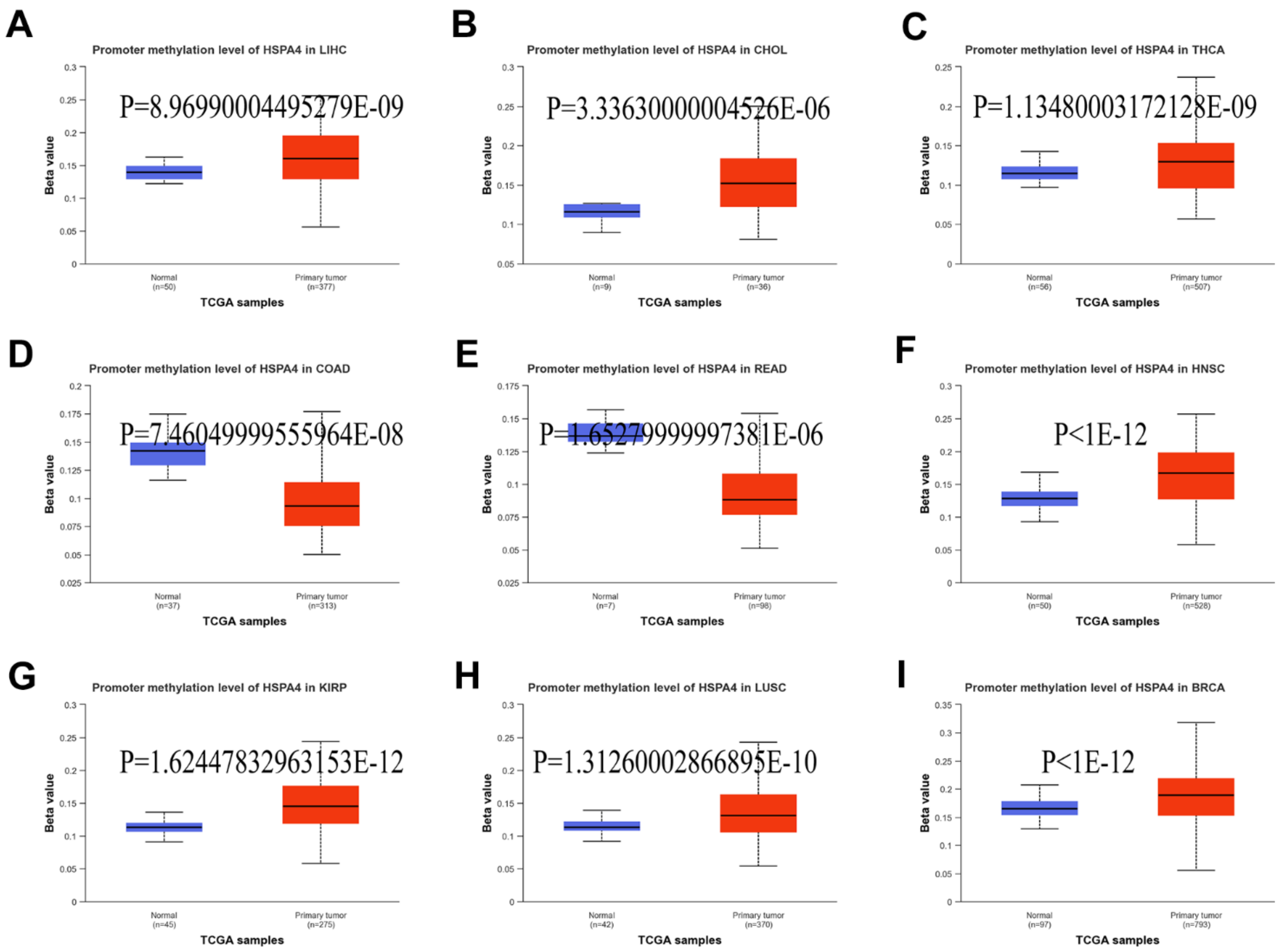

Supplementary Figure 2. Methylation levels of HSPA4 promoter in cancers. The UALCAN tool was employed to analyze the methylation values of HSPA4 in both normal and primary tumor tissues, including: (**A**) LIHC, (**B**) CHOL, (**C**) THCA, (**D**) COAD, (**E**) READ, (**F**) HNSC, (**G**) KIRP, (**H**) LUSC and (**I**) BRCA.

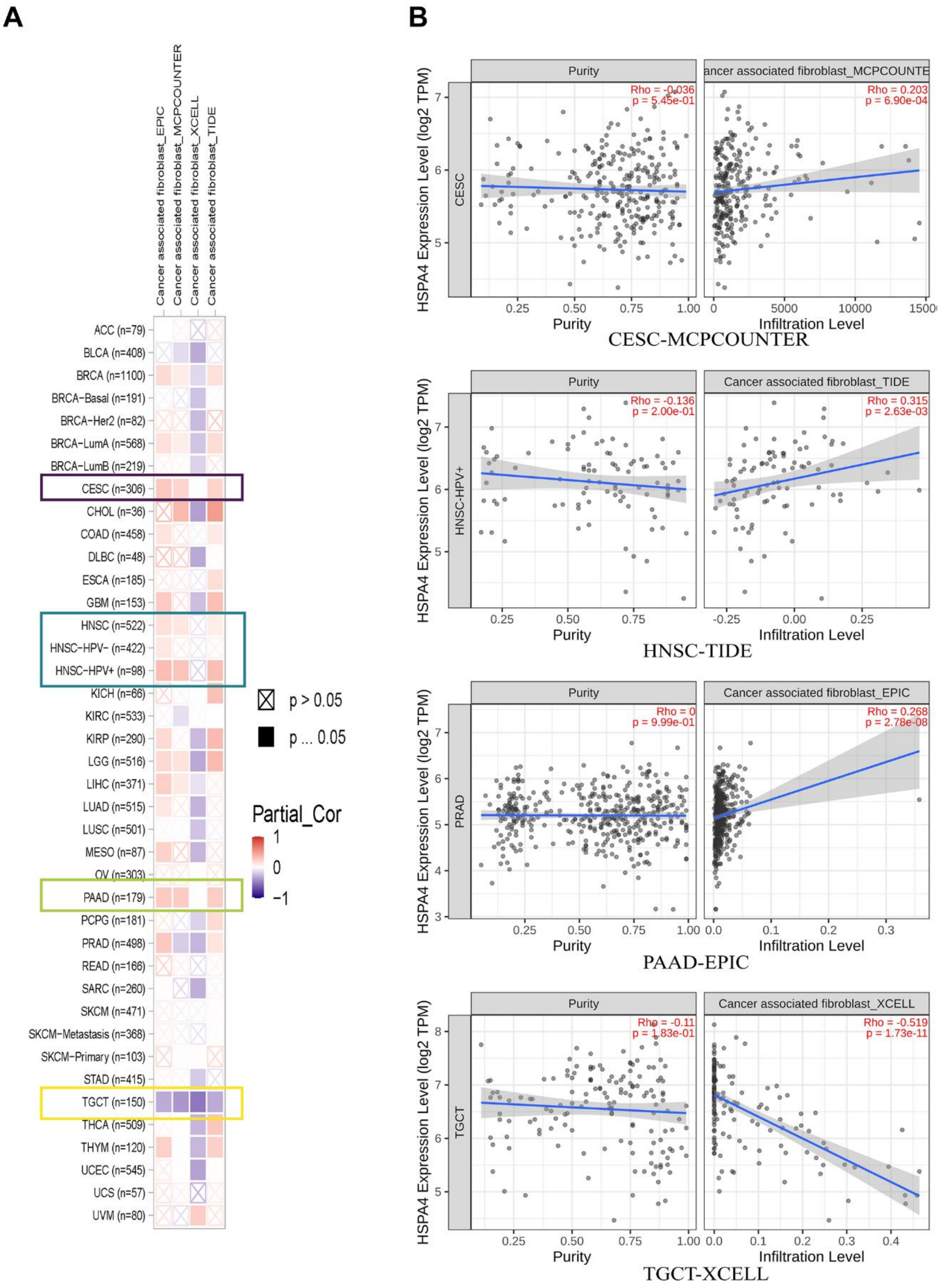

Supplementary Figure 3. Immune infiltration analysis. In a comprehensive analysis of cancers across TCGA, various algorithms were employed to investigate the potential correlation between SND1 gene expression levels and the infiltration levels of cancer-associated fibroblasts. (**A**) Pan-cancer heatmap generated using four distinct algorithms. (**B**) Scatter plot produced using one of the specified algorithms, illustrating the respective correlation.

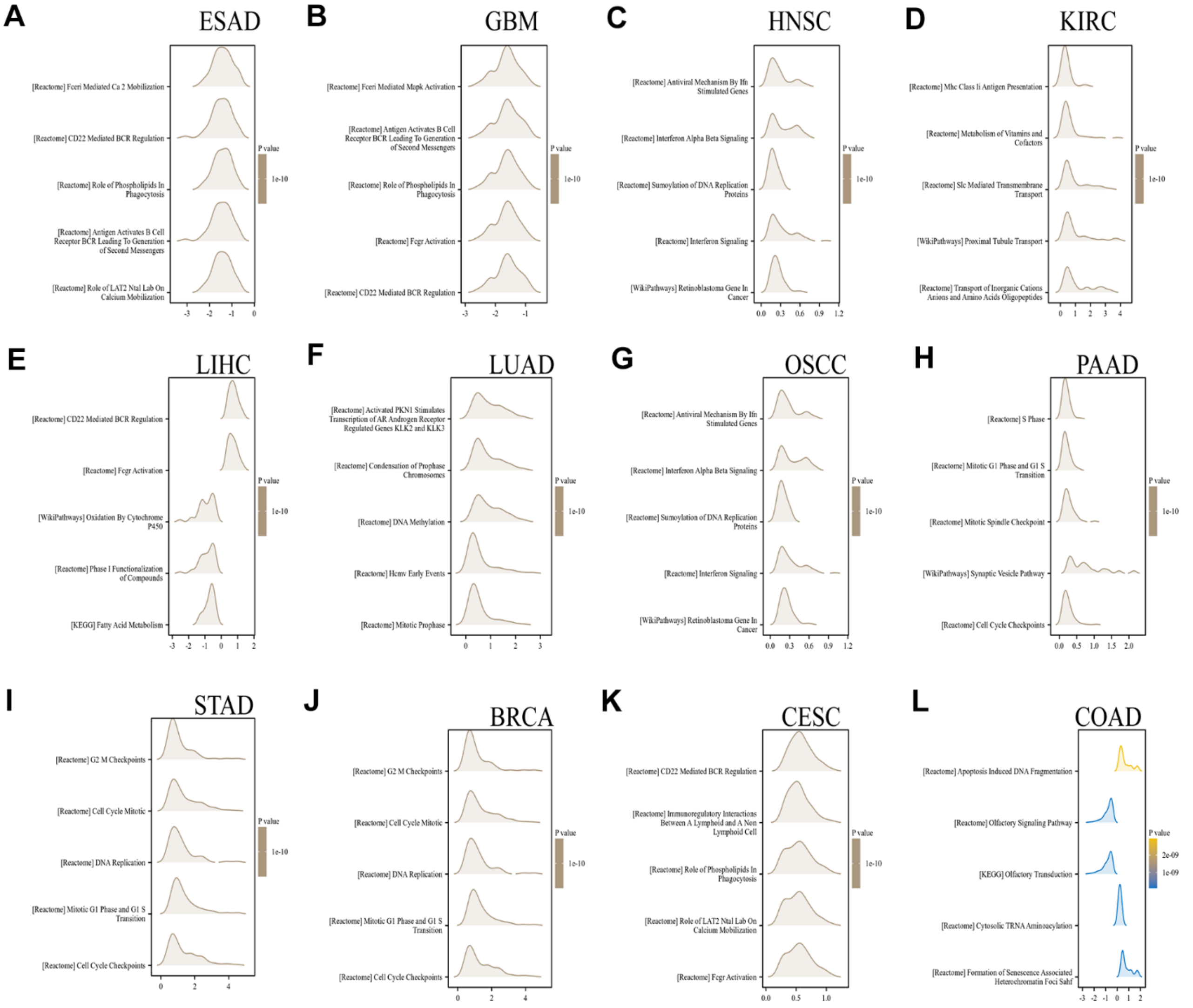

Supplementary Figure 4. GSEA enrichment analysis. GSEA for HSPA4 expression was conducted in (**A**) ESAD, (**B**) GBM, (**C**) HNSC, (**D**) KIRC, (**E**) LIHC, (**F**) LUAD, (**G**) OSCC, (**H**) PAAD, (**I**) STAD, (**J**) BRCA, (**K**) CESC, and (**L**) COAD. The y-axis represents a gene set, while the x-axis depicts the logFC distribution of core molecules within each gene set.
