# Supplementary material for: Unveiling the potential of HSPA4: a comprehensive pan-cancer analysis of HSPA4 in diagnosis, prognosis, and immunotherapy
Source: Aging (Albany NY). 2024 Jan 31;16(3):2517–41. doi: 10.18632/aging.205496 (PMC10911360; doi:10.18632/aging.205496)
Supplement: Supplementary Table 1 [file aging-16-205496-s002.pdf]

SUPPLEMENTARY TABLE

Supplementary Table 1. Abbreviations of cancers.

Abbreviations	Full name
BRCA	Breast invasive carcinoma
BLCA	Bladder urothelial carcinoma
CESC	Cervical squamous cell carcinoma and endocervical adenocarcinoma
COAD	Colon adenocarcinoma
CHOL	Cholangiocarcinoma
DLBC	Diffuse large B-cell lymphoma
ESAD	Esophageal Adenocarcinoma
ESCA	Esophageal carcinoma
GBM	Glioblastoma multiforme
HNSC	Head and neck squamous cell carcinoma
KIRC	Kidney renal clear cell carcinoma
KICH	Kidney chromophobe
KIRP	Kidney renal papillary cell carcinoma
LGG	Brain Lower Grade Glioma
LIHC	Liver hepatocellular carcinoma
LUAD	Lung adenocarcinoma
LUSC	Lung squamous cell carcinoma
OSCC	Oral squamous cell carcinoma
PAAD	Pancreatic adenocarcinoma
READ	Rectum adenocarcinoma
PRAD	Prostate adenocarcinoma
STAD	Stomach adenocarcinoma
THCA	Thyroid carcinoma
THYM	Thymoma
TGCT	Testicular germ cell tumors
UCEC	Uterine corpus endometrial carcinoma
